# TMS of primary motor cortex with a biphasic pulse activates two independent sets of excitable neurones

**DOI:** 10.1016/j.brs.2018.01.001

**Published:** 2018

**Authors:** Martin Sommer, Matteo Ciocca, Raffaella Chieffo, Paul Hammond, Andreas Neef, Walter Paulus, John C. Rothwell, Ricci Hannah

**Affiliations:** a Department of Clinical Neurophysiology, University Medical Center Göttingen, University of Göttingen, Robert-Koch-Str. 40, 37075 Göttingen, Germany; b Sobell Department of Motor Neuroscience and Movement Disorders, UCL Institute of Neurology, Queen Square, London WC1N 3BG, United Kingdom; c Scientific Institute Vita-Salute University San Raffaele, Neurological Department, Experimental Neurophysiology Unit, INSPE – Institute of Experimental Neurology, Milan, Italy; d Bernstein Focus Neurotechnology Göttingen, Germany

**Keywords:** Transcranial magnetic stimulation, Pulse shape, Monophasic, Biphasic, Motor cortex

## Abstract

**Background:**

Biphasic pulses produced by most commercially available TMS machines have a cosine waveform, which makes it difficult to study the interaction between the two phases of stimulation.

**Objective:**

We used a controllable pulse TMS (cTMS) device delivering quasi-rectangular pulse outputs to investigate whether monophasic are more effective than biphasic pulses.

**Methods:**

Temporally symmetric (“biphasic”) or highly asymmetric (“monophasic”) charge-balanced biphasic stimuli were used to target the hand area of motor cortex in the anterior-posterior (AP) or posterior-anterior (PA) initial current direction.

**Results:**

We observed the lowest motor thresholds and shortest motor evoked potential (MEP) latencies with initial PA pulses, and highest thresholds and longest latencies with AP pulses. Increasing pulse symmetry tended to increase threshold with a PA direction whereas it lowered thresholds and shortened latencies with an AP direction. Furthermore, it steepened the MEP input-output curve with both directions.

**Conclusions:**

“Biphasic” TMS pulses can be viewed as two monophasic pulses of opposite directions, each stimulating a different set of interneurons with different thresholds (PA < AP). At threshold, the reverse phase of an initially PA pulse increases threshold compared with “monophasic” stimulation. At higher intensities, the reverse phase begins to activate AP-sensitive neurones and increase the effectiveness of stimulation above that of a “monophasic” PA pulse. “Biphasic” stimulation with initially AP pulses is dominated at threshold by activation produced by the lower threshold reverse (PA) phase.

**Significance:**

The effects of biphasic stimulation are best understood as the summed output of two independent sets of directionally selective neural populations.

## Highlights

• Controllable pulse parameter TMS device was used to systematically modulate pulse shape.
• Pulse shape influenced primary motor cortex thresholds and input-output curves.
• Increasing pulse symmetry increased threshold with PA but lowered it with AP direction.
• Biphasic stimuli yield the summed output of directionally selective neural populations.

## Introduction

**Fig. 1:**
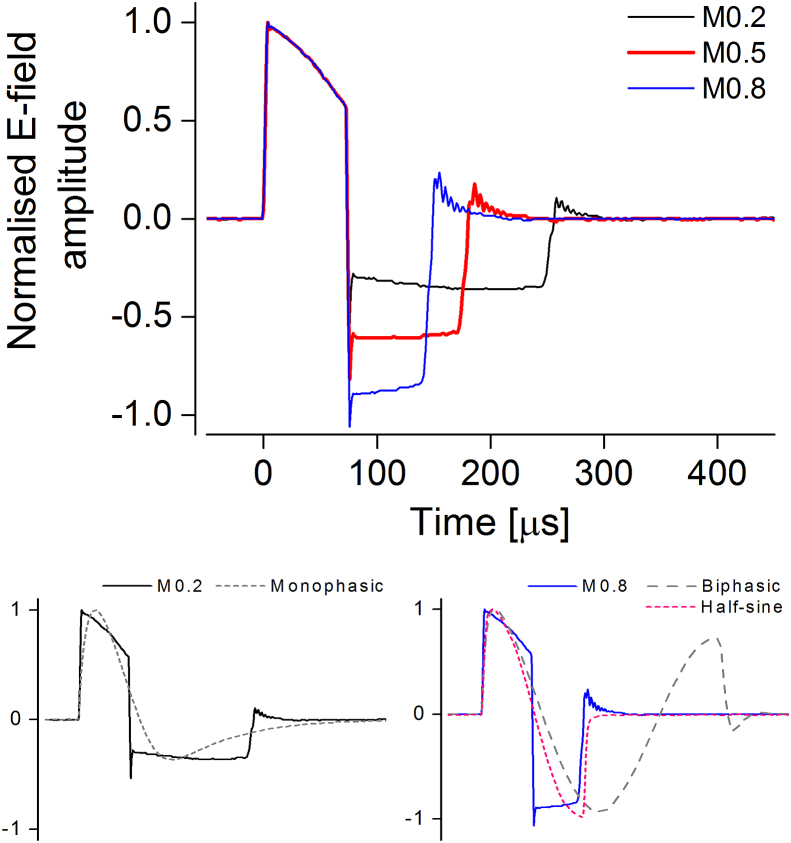
Types of TMS pulses used in this study. Current induced in a probe coil of 2 cm diameter held under the centre of a figure-of-eight-coil connected to a prototype cTMS device (Rogue Resolutions Ltd., Cardiff, United Kingdom), recorded and stored by an oscilloscope. So-called “positive monophasic” pulses with a pulse width of 75 μs and intensity of 20% of maximum stimulator output. Main figure illustrates cTMS pulses with distinct M-ratios: 0.2, 0.5 and 0.8, annotated in all figures as M0.2, M0.5 and M0.8, respectively. Amplitudes are normalised to the peak electric field recorded for each pulse. For comparison, conventional monophasic, half sine and biphasic pulse shapes are shown below. Note that the M0.8 pulse is more similar to a half sine than a biphasic pulse.

Transcranial magnetic stimulation (TMS) uses a magnetic field to induce electric currents in the brain, bypassing the resistance of skull and skin. Most commercially available TMS devices produce one of two charge-balanced pulse shapes: predominantly uni-directional (monophasic) pulse or bi-directional (biphasic) pulses (Fig. 1). Monophasic pulses are conventionally used for single pulse TMS in the assessment of corticospinal tract excitability and integrity [1]. Biphasic pulses are sometimes used for the assessment of corticospinal excitability [2,3], but most commonly for repetitive TMS (rTMS) protocols [[4], [5], [6]] because of the ability to achieve higher frequencies of stimulation [7].

Previously, commercially-available TMS machines tended to produce asymmetric biphasic pulses with a cosine waveform (Fig. 1), in which stimulation is dominated by the reverse phase, which is of equal amplitude but twice the duration of the initial quarter cycle phase. Less common are devices which produce a half sine pulse, consisting of two opposing quarter-cycle phases (Fig. 1), which more closely approximates a traditional biphasic pulse in that it is symmetrical with regard to duration and amplitude of the first and second phase [8,9] The present experiments used a new cTMS3 device [10], which can produce quasi-rectangular pulses and allows independent control of the duration and amplitude of each phase of a traditional, symmetrical biphasic pulse (Fig. 1). Biophysical models of axonal excitation show that monophasic stimuli are more effective than biphasic stimuli [11]. This has been confirmed in experiments on amphibian nerve [12] and on the cochlear nerve [13]. The reason is that some neural membranes appear to be able to integrate externally applied stimulus currents and voltage-gated ion channels are activated at a delay. Consequently the second phase of a biphasic pulse has time to reduce the effective strength of the initial phase and impair the activation process [11]. However, this may not apply to peripheral sensory nerves using bipolar stimulation with a proximal cathode and distal anode. At intensities just above threshold, biphasic pulses can produce activation at both the cathode and the anode. The explanation for this is debated but may be due to the sensitivity of sensory fibres to break excitation at the anode (e.g. Refs. [14,15]). At present it is unknown what happens during stimulation of cortical neurones using TMS since it has not been possible to investigate this issue in detail, because commercially available stimulators do not have the capacity to systematically change the duration/depth of the second phase relative to that of the first.

Nevertheless, stimulation of cerebral cortex is likely to be more complicated than in a peripheral nerve because the complex geometry of the folded surface gives neurones a variety of orientations with respect to the electric field [[16], [17], [18]]. In particular, it is well known that when stimulating the primary motor cortex the orientation of the current induced across central sulcus influences the activation of the corticospinal tract. Monophasic pulses applied at threshold produce earlier and less dispersed activity when the current is orientated approximately posterior-anterior (PA) across the central sulcus compared to anterior-posterior (AP) [19]. These differences are thought to reflect the activation of distinct excitatory synaptic inputs to corticospinal neurones each with a different preference for current direction, as well as a different threshold [20]. This means that when we use a biphasic pulse to activate cortex the reverse phase may not only cut short (and reduce) excitation produced by the initial phase, it will also activate a *different* set of neurones that are preferentially sensitive to the direction of the reverse phase. These factors could potentially explain the more complex pattern of corticospinal activation with asymmetric biphasic versus monophasic pulses [21]. This might also explain why the supposedly less effective biphasic pulse has a steeper MEP input-output (I/O) curve than a PA-directed monophasic pulse [22].

Elucidating the influence of pulse shape (monophasic/biphasic) and current direction (PA/AP) on corticospinal responses to TMS is critical to selecting optimal TMS parameters for different paradigms. For example, distinct sets of interneuron circuits in the cortex have different physiological properties, responding differentially to short-interval intracortical inhibition [23] and short-latency afferent inhibition [24]. Furthermore, differences have been shown in the response to rTMS protocols delivered with monophasic and asymmetric biphasic pulses [2,25]. We therefore used a novel controllable pulse parameter TMS (cTMS; [26]) device to modulate the relative amplitude of the first and second phases of a symmetrical pulse, exploring the effects on resting and active motor threshold, motor evoked potential (MEP) I/O curve, MEP latency, as well as contralateral silent period duration (cSP). We expected that activation of the lower threshold PA-sensitive elements with PA pulses would be less effective with symmetric biphasic compared with monophasic pulses. Predicting the outcome for higher threshold AP pulse types is more challenging, however, as there may be a trade-off between the reverse (PA) phase activating PA-sensitive elements and at the same time reducing effectiveness of the AP phase.

## Material and methods

We used a prototype cTMS machine (Brainsight cTMS3; (manufactured by Rogue Research, Montreal, Quebec, Canada. Supplied by Rogue Resolutions Ltd, Cardiff, United Kingdom; see also [26] for details) connected to a standard figure-eight-coil with an outer diameter of each wing of 70 mm (The Magstim Co. Ltd., Whitland, United Kingdom). In a pilot study, we used a single-loop search coil placed underneath the TMS coil and connected to a digital storage oscilloscope (SmartDS, Owon Technology Ltd, Kingston upon Thames, United Kingdom) to record “positive monophasic” waveforms generated by the cTMS device at a range of different “M-ratios”. The M-ratio refers to the ratio of the capacitor voltages which, in turn, controls the ratio of the E-field phases. We note that due to resistive losses the relationship between the capacitor voltages and E-field phases is imperfect, as can be seen the recorded E-fields for each M-ratio in Fig. 1.

In the main experiment, we studied 10 healthy subjects (mean age 29, range 24–43 years, 4 women) with no previous or current medical or neurological disease and no intake of CNS-active medication. They were selected from a group of 15 participants of a different study [27] for their relatively low motor threshold, because the cTMS device does not enable amplitude increases of more than 48% of maximum stimulator output (MSO) in all conditions studied here, which clearly limits the range of higher intensities that can be investigated.

We used a range of M-ratios (0.2, 0.5 and 0.8; where 0.2 reflects a more monophasic pulse and 0.8 a more biphasic pulse) with pulses of 75 μs pulse width, which is similar to the width of the initial component of many established TMS devices [28]. Pulses were delivered with both anterior-posterior (AP) and posterior-anterior (PA) oriented induced currents in the brain (i.e. perpendicular to the central sulcus). The order of all conditions (three M-ratios with two current directions) was randomized, with a single change in current direction by manually rotating the coil. For each pulse type, we used TMS over the optimal first dorsal interosseous representation of the right hand. The optimal position was defined as that eliciting the largest and most consistent MEPs when using a PA induced current and 0.2 M-ratio pulse, and was marked on a tight-fitting cap worn by the participants to ensure consistent coil placement. PA induced currents were produced by holding the coil so that the handle pointed approximately 45° postero-laterally. To induce currents in the AP direction in the brain, we rotated the coil by 180° in the horizontal plane so that the coil handle pointed antero-medially. The marks on the cap ensured that the coil centre position relative to the subject's head was consistent for AP and PA orientations.

We recorded electromyographic activity from the first dorsal interosseous muscle of the dominant hand using Ag/AgCl cup electrodes in a belly-tendon montage. The raw signal was amplified ( × 1000) and bandpass filtered (2 Hz–2 kHz) (D360, Digitimer Ltd, Welwyn Garden City, United Kingdom). Signals were sampled at 5 kHz (CED Power1401; Cambridge Electronic Design, Cambridge, United Kingdom) and analysed off-line using Signal version 5.07 (Cambridge Electronic Design, Cambridge, United Kingdom).

Motivated by the I/O curve recordings using different pulse widths by the Duke group who developed the cTMS [26], we recorded the MEP I/O curves applying intensities starting at 11% MSO (the lowest intensity technically achievable) and increasing to 48%MSO (the maximum intensity available for all types of stimuli), recording one MEP for each percent point of intensity increase. We refrained from randomizing the intensities, since this would have been difficult to implement using the manual intensity controls of the cTMS, and its putative advantage [29] has been questioned [30]. Each I/O curve (AP 0.2, AP 0.5, AP 0.8, PA 0.2, PA 0.5, and PA 0.8) was recorded twice with the target muscles at rest. After finishing all these recordings at rest, each I/O curve was recorded a third time, again in random order, with the FDI slightly active at about 10% of maximum voluntary EMG amplitude.

Following the I/O curve recordings and a 10 min break, we determined the resting motor threshold (RMT) as well as the active motor threshold (AMT) by reducing the stimulus intensity stepwise until only 3 out of 6 consecutive pulses induced MEP amplitudes of at least 50 μV with the hands at rest, or 250 μV during slight (about 10% maximum) voluntary abduction of the right index.

### Data analysis

All data were analysed using Statview 5.0. All *post-hoc t*-tests were uncorrected for multiple comparisons [31]. For motor threshold analysis, we calculated a repeated measures ANOVA with *voluntary activation* (two levels: AMT and RMT), *current direction* (two levels: AP and PA), and *M-ratio* (three levels: 0.2, 0.5 and 0.8) as independent variables. *Post-hoc t*-tests were performed on the overall threshold data after collapsing across AMT and RMT.

For I/O curve analysis, we adjusted for differences in motor threshold by repeating the analysis above, only taking into account the recordings with MEP amplitudes clearly exceeding the pre-TMS baseline at visual inspection of each trace. This analysis was confined to the first 11 intensity increases above the weakest intensity level with a discernible MEP. We entered the data into a repeated-measures ANOVA with intensity (7 levels: 0-6% above threshold), *voluntary activation* (two levels: rest and active), *current direction* (two levels: AP and PA), and *M-ratio* (three levels: 0.2, 0.5 and 0.8) as independent variables. While Fig. 4 shows all 11 levels of intensity, the analysis of intensity was confined to the first 7 intensities because data from some individuals were missing at higher intensity levels due to those participants having high motor thresholds meaning that the absolute starting intensity was close the maximum output of the stimulator. *Post-hoc t*-tests were performed on the overall data collapsed across AP and PA, rest and active, and all intensities to compare the effects of *M*-ratio on I/O curves.

**Fig. 2:**
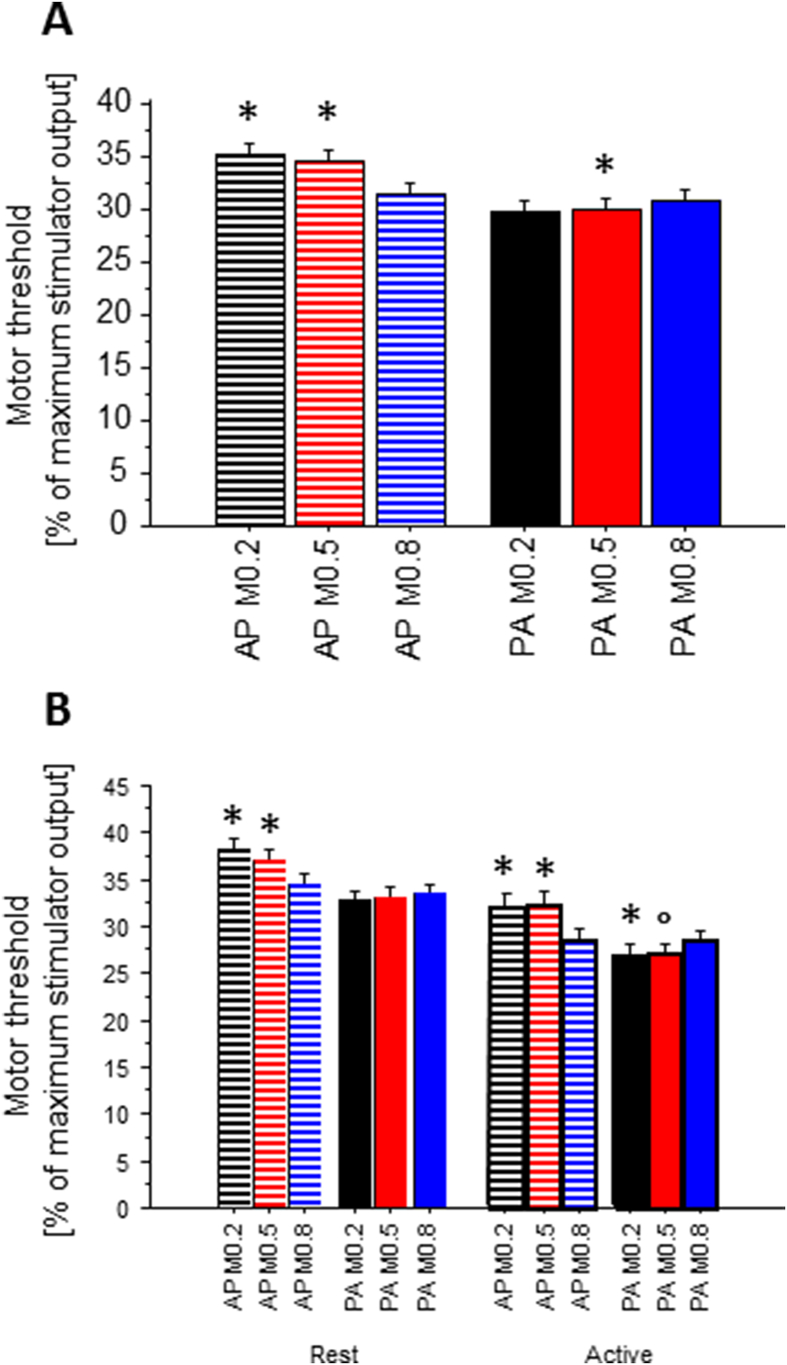
Motor threshold (mean of AMT and RMT) with anterior-posterior (AP) or posterior-anterior (PA) current direction in the brain, with varying degrees of pulse amplitude symmetry (M), mean ± SE. A, data across all levels of muscle activity (rest and active); B, each level of muscle activity depicted separately. Symbols indicate a difference with respect to the M-ratio 0.8 within each current direction: (*, p < .05; °, p = .057).

**Fig. 3:**
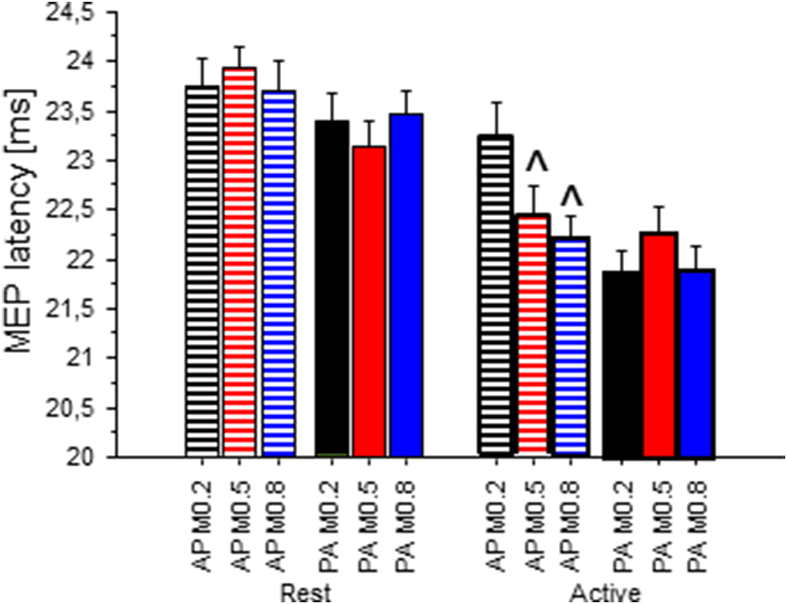
MEP latency. Average values from the five lowest intensity sweeps with discernible MEPs of the first MEP I/O curve recording, mean ± SE. Each level of muscle activity (rest and active) depicted separately. Symbols indicate a significant difference with respect to the M-ratio 0.2 (˄, p < .05) within each current direction.

**Fig. 4:**
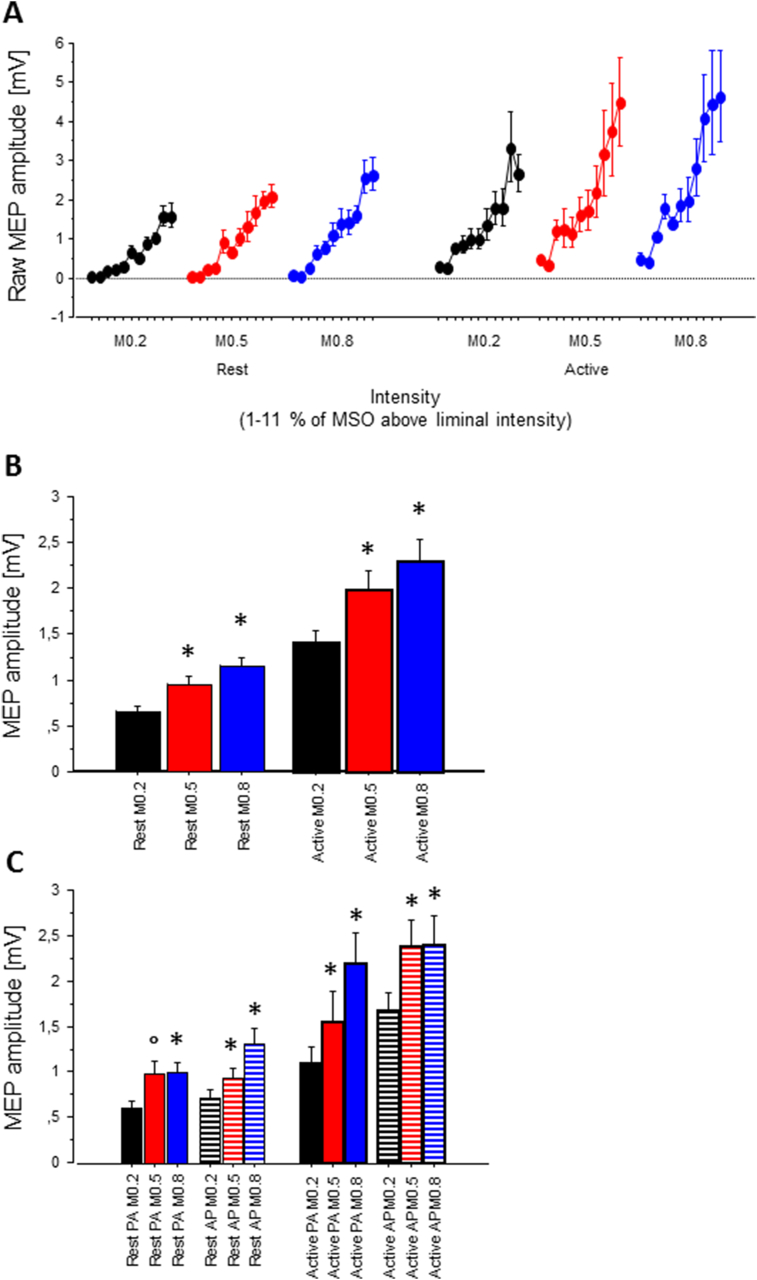
MEP I/O curve A, MEP I/O curve, illustration of the interaction of M-ratio by intensity, separated by current direction; B, illustration of the main effect of M-ratio, C, separated by current direction; A to C, data at rest and during activity displayed separately, mean ± SE. A-C, data from 1 to 11 % of MSO above the first intensity where a clearly discernible MEP could be detected. The analysis of intensity confined to the 7 weakest intensities (see text). Symbols indicate a difference with respect to the M-ratio 0.2 (*, p < .05 and °, p = .055).

For cSP duration, we determined the raw silent period duration from the active recordings at the time between MEP onset and the resumption of volitional muscle activity after the MEP. First EMG data from each sweep were rectified and the mean and standard deviation of the pre-TMS EMG activity (80 ms prior to TMS) was calculated. The onset of MEPs and volitional muscle activity were defined as the point when EMG activity increased by 2 standard deviations above the mean. cSP values were entered in a repeated-measures ANOVA with *current direction* (two levels: AP and PA), *M-ratio* (three levels: 0.2, 0.5 and 0.8) and *intensity* (13 levels: 11% MSO to 23%MSO) as independent variables. Sweeps without a discernible cSP were discarded, such that measurements started at the lowest individual intensity where a cSP could be measured. The ANOVA was limited to the lower 13 intensity levels available under all conditions of stimulation. We conducted a similar analysis after normalizing the cSP duration to the corresponding peak-to-peak MEP amplitude (Orth and Rothwell, 2004). We analysed 11 and 13 intensity levels for MEPs and cSP, respectively, because (1) cSPs can sometimes be detected without an MEP, and (2) the available range of intensities was bigger for cSP because they were obtained during activity which lowers threshold compared to rest.

We measured the MEP latency from the first five sweeps with discernible MEP amplitudes of the first I/O curve recording at rest, as well as from the I/O curve recorded during voluntary activity. We entered the latencies in a repeated-measures ANOVA with *current direction* (two levels: AP and PA), and *M-ratio* (three levels: 0.2, 0.5 and 0.8), muscle activity (two levels: rest and active) and sweep (five levels: sweep 1 to 5) as independent variables. We expected latency differences to be clearer during voluntary activity than during rest, because the higher stimulus intensities needed to evoke a response at rest recruit a mixture of inputs to corticospinal neurones [19,32,33]. We therefore ran similar planned ANOVAs separately for resting and active muscle conditions.

## Results

### Oscilloscope recordings

Oscilloscope recordings are shown in Fig. 1. They indicate that the symmetry of the induced current waveform gradually increases from a rather monophasic pulse waveform (M-ratio 0.2) to a rather symmetrical, more biphasic waveform (M-ratio 0.8).

### Recordings in humans

#### Side effects

No side effects of TMS were found.

#### Motor threshold and MEP latencies

For an initial PA direction, biphasic pulses (M-ratio = 0.8) had higher motor thresholds than monophasic pulses (M-ratio = 0.2). The opposite was true for pulses with an initial AP direction: they had lower thresholds when the pulse was biphasic compared with monophasic.

**Supplementary Fig. 1:**
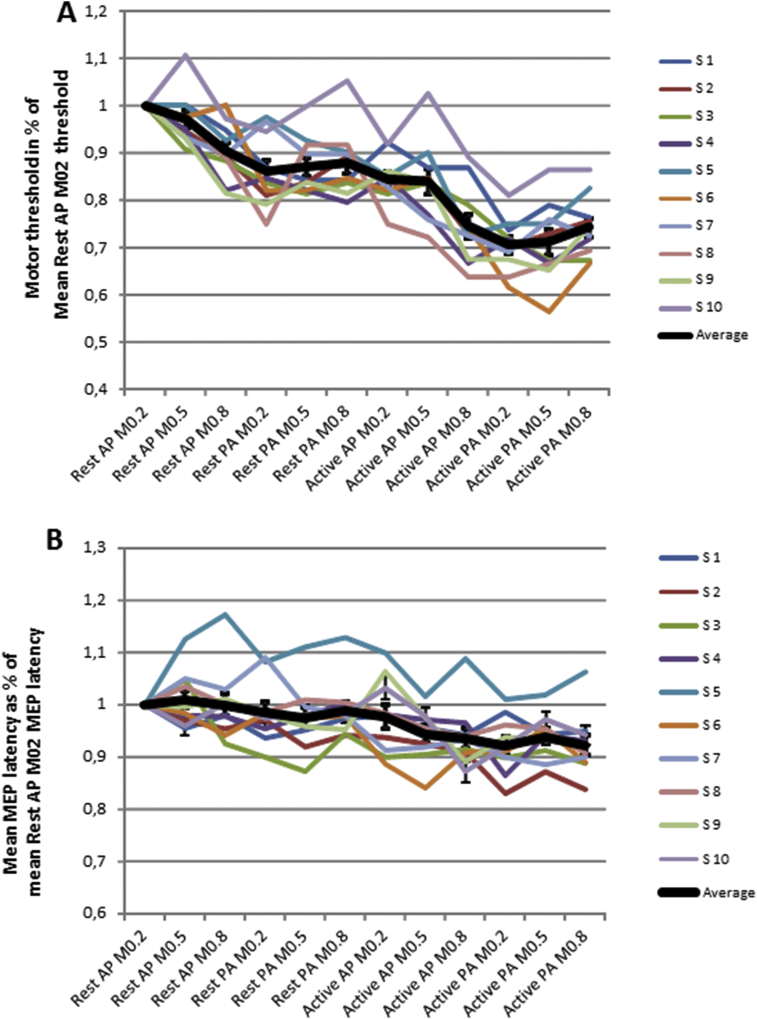
: Individual results for motor threshold (A) and MEP latency (B) normalised to the individual value in the condition with the target muscle at rest, current direction AP, M-ratio of 0.2. Thick line, mean ± SE.

In the statistical analysis, we observed main effects of voluntary activation (repeated-measures ANOVA, F_1, 9_ = 160.26, p < .0001), current direction (F_1, 9_ = 39.56, p < .0001) and M-ratio (F_2, 18_ = 6.36, p = .0082), as well as an interaction of current direction × M-ratio (F_2, 18_ = 22.59, p < .0001, Fig. 2). There were no other interactions. *Post-hoc t*-tests indicated significantly higher thresholds with the target muscle at rest than during voluntary activity and with AP versus PA current direction. Across levels of voluntary activation, with PA current direction, M-ratios of 0.2 and 0.5 yielded lower thresholds than the M-ratio of 0.8, whereas with AP current direction, M-ratios of 0.2 and 0.5 produced greater thresholds than the M-ratio of 0.8. Individual results are visualized in Supplementary Fig. 1a.

MEP latencies were shorter during activity than at rest, and were shorter if the pulse had an initially PA direction. However, during activation, the latency of biphasic AP pulses (M-ratio = 0.8) dropped to equal that of initially PA pulses.

The statistical analysis showed that there was a main effect of voluntary activation (repeated-measures ANOVA, F_1, 9_ = 31.3, p < .0001) and effect of current direction (F_1, 9_ = 10.02, p = .011), with PA pulses inducing shorter latencies than AP pulses. There was no main effect of M-ratio and of sweep (Fig. 3). Separate planned ANOVAs confirmed an interaction of current direction × M-ratio during voluntary activity (F_2, 18_ = 3.6, p = .047), but not at rest (repeated-measures ANOVA, F_2, 18_ = 0.6, p = .56). Individual results are shown in Supplementary Fig. 1b.

#### MEP I-O curve

In the threshold-adapted analysis, there was no difference in the slopes of the curves at rest compared with during activity. As reported by others [8,22], the curves were overall steeper for biphasic (M-ratio = 0.8) than monophasic pulse shapes (M-ratio = 0.2).

The repeated-measures ANOVA showed that all main effects were significant: voluntary contraction (F_1, 8_ = 30.53, p = .0006), current direction (F_1,8_ = 11.95, p = .009), intensity (F_6,48_ = 25.55, p < .0001) and M-ratio (F_2, 16_ = 10.20, p = .0014). The ANOVA also confirmed the interaction of M-ratio × intensity (F_12, 96_ = 1.94, p = .039; Fig. 4b), indicating that I-O curves were steeper for biphasic stimulation. Post-hoc t-tests confirmed shallower I/O curves with M-ratio 0.2 than with the other M-ratios. However, the interaction of current direction × M-ratio (F_2, 16_ = 3.77, p = .046; Fig. 4c), suggested that this effect was more prominent for PA versus AP currents. Finally, there were also interactions of voluntary activity × intensity (F_6, 48_ = 2.46, p = .037), and voluntary activity × current direction × M-ratio (F_2, 16_ = 4.22, p = .034). The current analyses were restricted to the first 7 intensity levels where complete data sets from all individuals were obtained. However, we note that inspection of the complete curves (including higher intensity levels where we know that not every participant contributed data) appears to indicate that the I-O curves are composed of an initial shallow slope accompanied by an inflection producing a steeper slope at higher intensities (e.g. intensities producing ∼1 mV MEP) (Fig. 4a). This is more noticeable for 0.2 M-ratio pulses compared to 0.8 M-ratio pulses.

#### Contralateral silent period

**Fig. 5:**
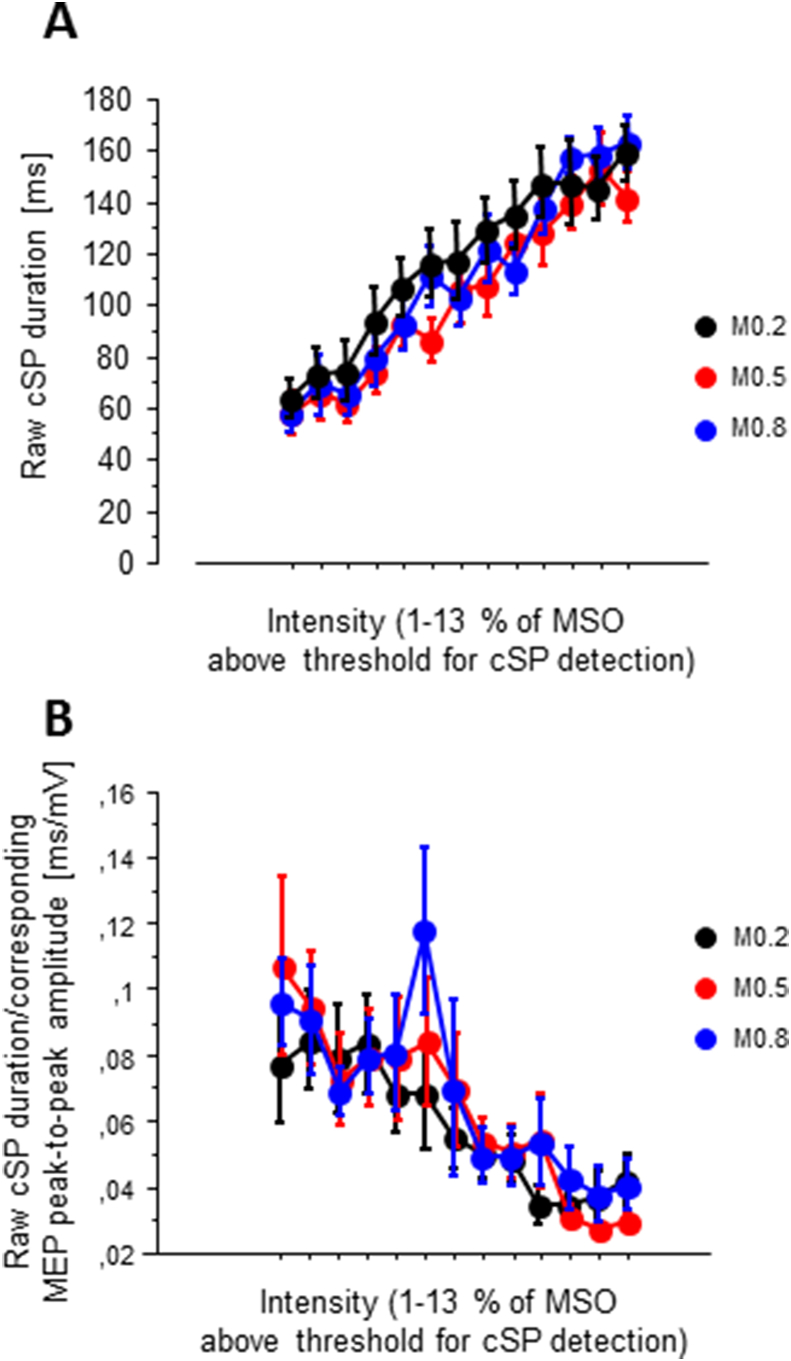
cSP I/O curve cSP durations recorded with stimulus intensity increasing in steps of 1 % maximum stimulator output, starting at the intensity where in each individual a first cSP was discernible. At each intensity level, average obtained from 10 healthy subjects. Mean ± SE, data from AP and PA current direction pooled. A, raw cSP duration; B, cSP duration divided by the corresponding peak-to-peak MEP amplitude.

Apart from a main effect of intensity (repeated-measures ANOVA, F_12, 84_ = 35.7, p < .0001, Fig. 5), no other significant main effects or interactions were found. Normalizing the cSP duration to the corresponding peak-to-peak MEP amplitude did not change this result substantially showing a main effect of intensity (repeated-measures ANOVA, F_12, 84_ = 7.16, p < .0001) but no interaction and only trend towards an effect of current direction (F_1, 7_ = 4.37, p = .075) (Fig. 5b).

## Discussion

As we argue below the present results suggest that the reverse phase of a TMS pulse can have two different effects on the activation of synaptic inputs to corticospinal neurones. One is to oppose ongoing membrane depolarisation and thus impair activation of excitatory inputs targeted by the initial phase. The result is that net synaptic input to the corticospinal neurones is reduced and threshold is higher than for monophasic pulses. The second effect of the reverse phase is to recruit an additional population of excitatory inputs that are sensitive to oppositely directed current. This increases net synaptic input to the corticospinal neurones above that provided by the initial phase of current. The result is that symmetrical biphasic pulses have a steeper slope of MEP recruitment. The relative importance of these two (opposing) effects depends on the threshold difference between activation of AP- and PA-sensitive neurones.

### MEPs evoked by PA-directed pulses

In line with previous results documented with traditional monophasic pulses [34,35], the most monophasic (M-ratio 0.2) PA pulse here was found to have lower threshold and a shorter MEP onset latency than the monophasic (M-ratio 0.2) AP pulse. These data are consistent with the idea that PA and AP pulses recruit distinct sets of excitatory synaptic inputs to corticospinal neurones [20]. For PA pulses, increasing the amplitude of the reverse phase (AP-directed) to create a more biphasic pulse (M-ratio 0.8) produced a small but significant increase in motor threshold and had no effect on the latency of responses. We interpret this as showing that the recruitment of the low threshold PA-sensitive inputs was impaired by the second phase of the biphasic pulse, as would be predicted based on responses of auditory nerve to monophasic and symmetrical biphasic pulses [13]. One explanation is that the neural elements activated by monophasic AP pulses have higher thresholds than those recruited by PA pulses, and that the reverse phase of biphasic PA pulses would be insufficient to recruit the AP-sensitive inputs and so these were unlikely to have contributed to the generation of threshold MEPs. An alternative explanation is that the two directions excite different sets of axons that have the same threshold, but different strengths of excitatory synaptic connections with pyramidal neurones. Both interpretations are consistent with the comparison of monophasic PA pulses and half sine initially PA pulses in an earlier study [8].

What then would explain the steeper I/O curves generated by the more biphasic pulses compared to the most monophasic pulse? The very initial part of MEP I/O curves is probably subject to the same detrimental effects of the reverse phase of a biphasic PA pulse, which impairs the activation of PA-sensitive inputs to corticospinal neurones. However, at higher stimulus intensities, the reverse phase of the pulse may be of sufficient amplitude to recruit the higher threshold AP inputs, thus increasing the overall excitatory synaptic input to the corticospinal neurones. This in turn could explain the steeper I/O curves of biphasic versus monophasic PA pulses, if we assume that the later arriving volleys produced by the reverse phase of the biphasic stimulus summate readily with the earlier volley produced by the initial PA phase.

A caveat to our interpretation above is that we assume that the amplitude of the second phase of monophasic M-ratio 0.2 pulses is below rheobase (∼10%MSO; [10]) for MEPs elicited at threshold, and is therefore unlikely to exert any influence despite its long duration. At higher intensities the amplitude of the second phase might just exceed rheobase and exert a small effect similar to that of the more biphasic pulses.

### MEPs evoked by AP-directed pulses

The situation for initially AP-directed pulses was quite different. Increasing the relative amplitude of the reverse (PA-directed) pulse phase, from an M-ratio of 0.2–0.8, had two prominent effects on MEPs generated at threshold. Motor thresholds and MEP onset latencies both decreased, such that values for the biphasic (M-ratio 0.8) AP pulse were similar to those obtained with monophasic (M-ratio 0.2) PA pulse. We speculate that this occurs because higher stimulus intensities are required to recruit AP-sensitive inputs compared to PA-sensitive inputs [19]. Thus when the initial and reverse phases of a biphasic pulse are approximately equal in duration and amplitude, i.e. symmetrical, it is the reverse PA phase that is responsible for recruiting the initial corticospinal excitation. As the intensity increases, the initial AP phase probably begins to recruit activity which increases the effectiveness of stimulation still further. Finally, it is possible that the second phase of the AP pulse impaired the activation of AP-inputs but any possible effect is made undetectable by the recruitment of the shorter latency PA inputs.

### Comparison with descending volleys recorded in the spinal epidural space

Recordings of the volleys evoked by TMS over the primary motor cortex (I-waves) showed that monophasic PA pulses preferentially induce early I-waves, but AP currents preferentially recruit later I-waves, suggesting different sites of action within the primary motor cortex by these two current directions [23,24,35]. With asymmetrical biphasic pulses in either AP or PA direction (referring to the direction of the initial phases), the I-wave pattern was reported to be variable and not always consistent with the expected dominant second phase of the pulse [21]. The present results go some way to explaining these inconsistencies by showing that the second phase is not in fact always dominant in the case of symmetrical biphasic pulses, and that these pulses can have a range of effects on the recruitment of different sets of interneurons. The net effect of these processes in an individual likely depends on individual interneuronal physiology and anatomy, and this may explain the differences in I-wave recruitment observed. Nevertheless, the data here are consistent with the notion of two completely separate interneuronal circuitries with different thresholds and no prominent interaction between them. An alternative view could be that layer 2/3 or layer 5 pyramidal neurones are stimulated along their long axis in two opposite directions [2]. This would be possible if the motor cortex is assumed to be allocated and maximally stimulated in the anterior wall of the central sulcus [36,37] and if an alternative model of I-wave generation is presumed [38]. In this alternative view, PA and AP latency differences could be accounted for by assuming that antidromically activating a cortical column via axons or via the dendritic trees might not immediately (re-)fire an action potential.

### Cortical silent period

The current protocol produced relatively short cSP durations, which did not appear to be affected by current direction and M-ratio. Previous research had reported that a given intensity of stimulation (expressed in %AMT) produced a shorter cSP for monophasic PA stimulation than for monophasic AP or biphasic PA stimulation [22]. The differences in that study were eliminated when the cSP duration was normalised to the size of the MEP, to account for differences in the I/O curves, suggesting that the generation of the MEP and cSP share a similar mechanism [22]. The authors in fact proposed that the cSP was generated by form of recurrent inhibition of corticospinal neurones activated by the TMS pulse.

Given the greater size of MEPs generated by biphasic versus monophasic pulses in the present I/O curves, we might have expected biphasic pulses to be associated with greater cSP durations when expressed in absolute terms. This was not the case, as cSPs were similar when expressed in both absolute and relative terms. When considered alongside previous literature, it seems that the production of MEPs and cSPs may share some, but not all mechanisms. Additionally, the difference in MEP amplitudes for the three M-ratios was never very large and may have been too small to generate reliable differences in cSP duration. This limits the conclusions that can be drawn from the present data when comparing to datasets exploring longer SP durations and a larger range of stimulus intensities [22].

Another limitation is the small number of trials used to estimate motor thresholds and to generate the I/O curves within each individual. However, the impact of this should be countered somewhat by the focus on average data from all individuals. A further limitation is that measurements of MEP latencies were assessed at different intensities relative to each respective motor threshold (i.e. a fixed number of absolute intensities above motor threshold were used). This could have affected the changes in MEP latencies across the different M-ratios by virtue of the different slopes of the I-O curves. We suspect, however, that any effect is likely to be relatively small.

## Conclusions

Taken together, this systematic variation of the pulse shape and current direction allows us to conclude that the symmetrical biphasic pulse is composed of two monophasic pulses of opposite directions that activate two quite separate sets of neurones.

## Role of the funding sources

The funding sources had no involvement in the conduct of the research, the collection, analysis and interpretation of data; in the writing of the report; and in the decision to submit the article for publication.

## Declaration of interest

None declared.
